# Why humans evolved blue eyes

**DOI:** 10.3389/fpsyg.2025.1442500

**Published:** 2025-04-16

**Authors:** Paola Bressan

**Affiliations:** Dipartimento di Psicologia Generale, University of Padova, Padua, Italy

**Keywords:** eye color, mate choice, sexual selection, social selection, parental selection, greenbeards, biological ornaments, Fisher’s runaway

## Abstract

A surprising number of humans are equipped with a subpar eye model—featuring pale, colorful irides that are nowhere as good as the original dark ones at guarding the retina from sunlight and do, in fact, raise one’s risk of eye disease. Here I apply evolutionary theory to understand why. I propose that the allele for human blue eyes, which arose just once, managed to spread from one individual to millions at an astonishing speed because it is a greenbeard. “Greenbeards”—imaginary genes, or groups of genes, that produce both a green beard and a behavior that favors other bearers of a green beard—have been deemed exceedingly unlikely to show up in the real world. And yet, as individuals who prefer blue eyes are more inclined to mate with blue-eyed partners and invest in blue-eyed offspring, any blue-eye preference (whether random or arising from the bias for colorful stimuli shared by all recognition systems) becomes rapidly linked to the blue-eye trait. Thus, blue eyes gain an edge by working like a peacock’s colorful tail *and* a nestling’s colorful mouth: twice self-reinforcing, “double runaway” evolution via sexual and parental selection. The blue-eye ornament gene, by binding to a behavior that favors other bearers of the blue-eye ornament gene, is ultimately recognizing and helping copies of itself in both kin and strangers—and greatly prospering, just like theory predicts.

## 1 Blue eyes make a splash

A nearly similar taste for beautiful colours… runs through a large part of the animal kingdom.

— *Charles Darwin, On the Origin of Species*

When we were all Africans, we all had brown eyes. Blue eyes arose from one single mutation in one single individual (Eiberg et al., 2008) who lived in Europe or the Near East earlier than 14,000 years ago (Fu et al., 2016). This mutation turned partly off the ability of one of our genes to produce melanin, the pigment that darkens eyes, hair, and skin. Were it not for melanin, human eyes would be blue—and for the same reason why the sky is blue. Like the sky, the colored part of the eye, the iris, hosts a multitude of small particles that scatter the incoming light in all directions; but the wavelengths that are scattered best are the shortest, those that we perceive as blue (Bohren, 1988). Yet the iris also contains melanin, which primarily absorbs light and hence masks the color that would otherwise be displayed: more melanin, darker eyes (Wielgus and Sarna, 2005).

Under the African strong, unremitting sun, we maintained very dark eyes—likely because the melanin in the iris, by absorbing rather than letting through most of the light that strikes it, protects the sensitive retina at the back from the deleterious effects of ultraviolet radiation (Hu et al., 2008). Among the multiple genes that determine the amount of melanin, the most important is *OCA2* (whose malfunctioning produces albinism and depigmented eyes: Grønskov et al., 2007), which lies on chromosome 15. Its expression, however, is regulated by a single variation within a short sequence highly conserved across species, rs12913832, on the nearby gene *HERC2* (Eiberg et al., 2008; Sturm et al., 2008; Visser et al., 2012). The ancestral allele at rs12913832 stimulates melanin production, leading to dark eyes; the derived (i.e., mutated, non-ancestral) allele reduces melanin production fivefold (Duffy, 2015), allowing the eyes to look blue. The latter allele behaves like a recessive trait, expressing itself only when it is inherited from both parents.

And here comes the mystery: for no evident reason, and in an evolutionary time span as short as a few thousand years, the number of people with blue eyes proceeded to expand from one to millions. Relative to the ancestral allele, the mutation that causes blue eyes is now present in proportions as impressive as 70 to 95% in northern Europe, and at lower but remarkable frequencies in southern Europe, Southwest Asia, and North Africa, down to a still appreciable 5 to 20% in Central and South Asia (Kidd et al., 2020).

The plot has thickened with the unsettling discovery that some ancient Europeans featured blue eyes along with the original dark, African-like skin (Olalde et al., 2014; Lazaridis et al., 2014; Fu et al., 2016). That goes to show that, counter to what we used to believe, light eyes *preceded* skin lightening (Günther et al., 2018; Hanel and Carlberg, 2020) and hence cannot be accidental byproducts of it. Indeed, although some genetic markers of iris color could have been selected for the effects they had on skin and hair pigmentation, this is plainly not the case for the rs12913832 mutation (Beleza et al., 2013): this allele shows evidence of having been under strong positive selection in the last 5,000 years specifically for its effect on eye color (Donnelly et al., 2012; Duffy, 2015; Edwards et al., 2016; Wilde et al., 2014).

### 1.1 Eye-color evolution by natural selection: the trouble with the “winter blues” hypothesis

There is, in fact, one way in which pale irides might turn into an asset. At the back of the eye, specialized cells rely on the amount of light that strikes them to regulate the main body clock, so as to align it with the rising and setting of the sun and prepare for day and night, summer and winter. Far from the equator, however, the adaptive winding down of activities in wintertime can develop into winter depression, or seasonal affective disorder (abbreviated, rather appropriately, to SAD). Winter depression manifests with low mood, lethargy, less motivation to be around others or have sex; in uncannily similar forms, it also shows up among our fellow creatures—from fish (Nakayama et al., 2020) to macaque (Qin et al., 2015). In ancestral immigrants newly arrived from better climates, the short, dim days and long nights of northern Europe may have induced winter depression for half the year.

Individuals with SAD are less sensitive to light during the winter and tend to improve markedly when exposed to bright light (Kaladchibachi and Fernandez, 2018; Lavoie et al., 2009; Okimura et al., 2021). For this reason, brown-eyed people are proner to winter depression (Goel et al., 2002; Workman et al., 2018) than blue-eyed ones, whose retinas get much more light to start with. It has indeed been suggested that the antidepressant properties of light irides at high latitudes might have been the motor behind the evolution of blue eyes (Goel et al., 2002; Sturm and Larsson, 2009; Workman et al., 2018).

Despite its clear conceptual appeal, this hypothesis raises at least two problems. First, at the latitudes where the earliest evidence of the blue-eye allele has been found (northern Italy and the southern Caucasus: Fu et al., 2016), pale irides would have been a rather mixed blessing—or would have rapidly become one, as the amount of incoming solar radiation increased after the end of the last glacial maximum (Clark et al., 2012). Given that melanin works both as a physical light screen and a chemical antioxidant (Boulton et al., 2001), in fact, a light eye is doomed to do a far poorer job than the original dark model in protecting the retina. Besides containing less melanin than brown ones, light irides even host a smaller amount of its black-brown type relative to the red-yellow one: the latter, under the attack of visible and ultraviolet light, may add insult to injury by acting as a pro-oxidant rather than as an antioxidant (Wakamatsu et al., 2008). Further worsening matters, the protective pigment in the central portion of the retina is less dense in light than in brown eyes (Hammond et al., 1996). Thus, it is no surprise that blue eyes come up as a risk factor for a variety of eye diseases. On account of their lighter irides, for example, white people are 18 times more likely than black ones to get uveal melanoma (Wakamatsu et al., 2008), the most common cancer of the eye and a malignant one at that. Individuals with blue eyes have higher odds of developing age-related maculopathy and macular degeneration (Mitchell et al., 1998) and losing their sight altogether as a result. And since blue irides let through 100 times more light than do dark-brown ones, light-eyed people suffer more from the adverse effects of glare, as the veil caused by bright areas degrade the visibility of details in darker areas of the scene (Van den Berg et al., 1991).

Second, and most crucially, the winter blues account requires that people whose seasonal depression is not mitigated by light irides leave fewer or less fertile offspring, but there is no indication they do or they might. Quite on the opposite, several lines of indirect evidence suggest that, as people migrated away from the equator and toward regions where food availability varied greatly during the year, the hibernation-like pattern of seasonal depression evolved precisely because it brought forth reproductive *advantages* (Davis and Levitan, 2005; Eagles, 2004). The psychological and behavioral impairments that accompany the dim days of fall and winter—including lowered enthusiasm for sex—remit, or even revert, during the bright days of spring and summer. So, women of childbearing age (among which SAD is *far* more common than in older women or in men: Davis and Levitan, 2005) would be more likely to become pregnant in early summer and give birth in early spring—when, with warmer temperatures and heftier food supplies ahead, babies would have higher chances of surviving (Eagles, 2004). Indeed, the very symptoms of SAD (diminished energy, activity, and sociality; increased appetite, weight gain, and sleep) correspond to, and would favor, the natural changes associated with pregnancy (Davis and Levitan, 2005).

Thus, the winter blues hypothesis would have to maintain that in other animals the seasonal behavioral changes that accompany seasonal climate changes happen for a very good reason, but in ancestral Europeans arose instead as a reproductive calamity or went systematically too far. Which amounts to saying that human light eyes evolved so as to counteract an orderly, robust, across-species seasonal behavioral pattern—while the pattern itself did not evolve at all but occurred, and has kept occurring for the last several thousand years, as an abnormal and maladaptive response to the environment. And yet even if such an argument made sense, the best that can be said of the winter blues hypothesis of light eyes evolution is that it is a vastly incomplete explanation of the facts: as we shall see, it accounts for hardly any of the specific empirical findings that will be presented in the remainder of this paper.

### 1.2 Eye-color evolution by sexual selection: the trouble with the “rare/novel color advantage” hypothesis

If it cannot be explained by ordinary natural selection, the prodigious ascent of blue eyes may have been driven by *sexual* selection: that is, by their carriers gaining some advantages over rivals “in battle or courtship,” being such advantages “in the long run greater than those derived from rather more perfect adaptation to their conditions of life” (Darwin, 1871). In short, potential mates with blue eyes must have been liked a great deal, for reasons that have as yet escaped us (Cavalli-Sforza et al., 1994; Duffy, 2015; Liu et al., 2013). The only such reason offered so far is a “rare color advantage” effect (later renamed “novelty” effect: Frost, 2006, 2014), an idea which has rapidly garnered much wider popularity than the winter blues one. The “rare/novel color advantage” hypothesis suggests that ancestral European women evolved unusual eye colors to catch the attention of potential husbands—men being a scarce commodity at the time, due to hunters’ harsh lifestyle in the inhospitable Eurasian tundra (Frost, 2006, 2014).

It seems to me that this proposal encounters a few difficulties, however. First, being rare or novel is not always ground for being preferred (see Janif et al., 2015); it might even, and every bit as easily, be ground for being avoided. For example, yellowish or exceedingly pale irides are less common than either brown or blue ones, yet one would be hard pressed to argue that, on account of their rarity or novelty, they are especially well liked. A lack of predilection for certain rare or novel eye colors, of course, would hardly be surprising if these signal health troubles, like do pink eyes—as those of some albinos, whose want of melanin lets the blood vessels at the back of the eye show through. Notably, the first chimpanzee infant with albinism sighted in the wild prompted violent fear in its conspecifics and was killed by them (Leroux et al., 2021); humans, alas, share not too dissimilar instincts (Benyah, 2017; see also Bressan, 2021c). Still, data collected in Italy show that, for instance, dark-brown irides—which entail no health disadvantage to speak of—are much scarcer than brown ones (17% vs. 40%: Bressan, 2024) and yet are liked significantly less (as we shall see in section 4).

Second, green or hazel eyes are way rarer than blue ones (Walsh et al., 2012; see also Figure 3 in Kidd et al., 2020). Yet there is evidence of intense selection for the blue-eye allele but not for alleles associated with green/hazel eyes (such as rs1800407: Donnelly et al., 2012, or the Celtic-ancestry marker rs12203592). These alleles’ main effect, oddly enough, appears to be that of raising the probability that carriers of two blue-eye alleles express *blue* eyes (Duffy, 2015; Sturm et al., 2008)—while lowering the probability that carriers of one blue-eye allele express brown eyes (Duffy, 2015). The “rare/novel color advantage” hypothesis makes much of eye color diversity, with no particular emphasis on blue. Yet the best visible eye color variations stem not from specific “color alleles,” but from two rather more prosaic contingencies. The first is rs12913832 heterozygosis (the presence of one “blue” and one “brown” allele), which results in variable amounts and qualities of melanin, sometimes very unevenly spread across the iris (Walsh et al., 2012). The second is a host of small inhomogeneities in the density and distribution not only of the pigment itself but also of collagen fibrils, that produce different shades of color by scattering the light around in all sorts of ways (Sturm and Larsson, 2009). Such variability reflects the vast diversity of cells in the iris and the complexity of this tissue—whose formation involves nearly 2,700 genes (Sturm and Larsson, 2009)—and hence may not require an explanation based on rare-trait sexual selection. Further corroborating this point, the frequency of eye colors other than blue and brown is far higher in populations that experienced genetic admixtures (Salvoro et al., 2019), such as some southern European ones, than it is in northern Europe (for example, it is 25% in Italy but less than 7% in Norway and Estonia)—although northern Europe is precisely where sexual selection is supposed to have had the freest rein. The greater this freedom, the more the mating market appears to have been saturated with blue, rather than diversified with all manner of eye-catching hazels and reds and oranges and yellows and greens and violets.

Third, in hunter-gatherers inhabiting northern Europe 8,000 years ago, blue eyes appear to have been already far more frequent than brown ones; in fact, all specimens found so far had blue eyes (Mathieson et al., 2015a). Yet, one would expect a “rare/novel color advantage” effect to have started favoring the less common brown eyes well before blue ones became fixed. Also belying the merits of rarity, modern Britons’ genomes have revealed that blue eyes have been under *increasingly* strong positive selection even over the last 100 generations, that is for the past 2–3,000 years (Field et al., 2016). Indeed, the pervasiveness of the allele specifically associated with blue eyes is among the strongest signatures of recent selection in humans (Field et al., 2016; Mathieson et al., 2015a). In this paper, I propose an explanation for such a rapid, unexpected ascent.

## 2 Blue eyes as peacock tails

**Figure 1:**
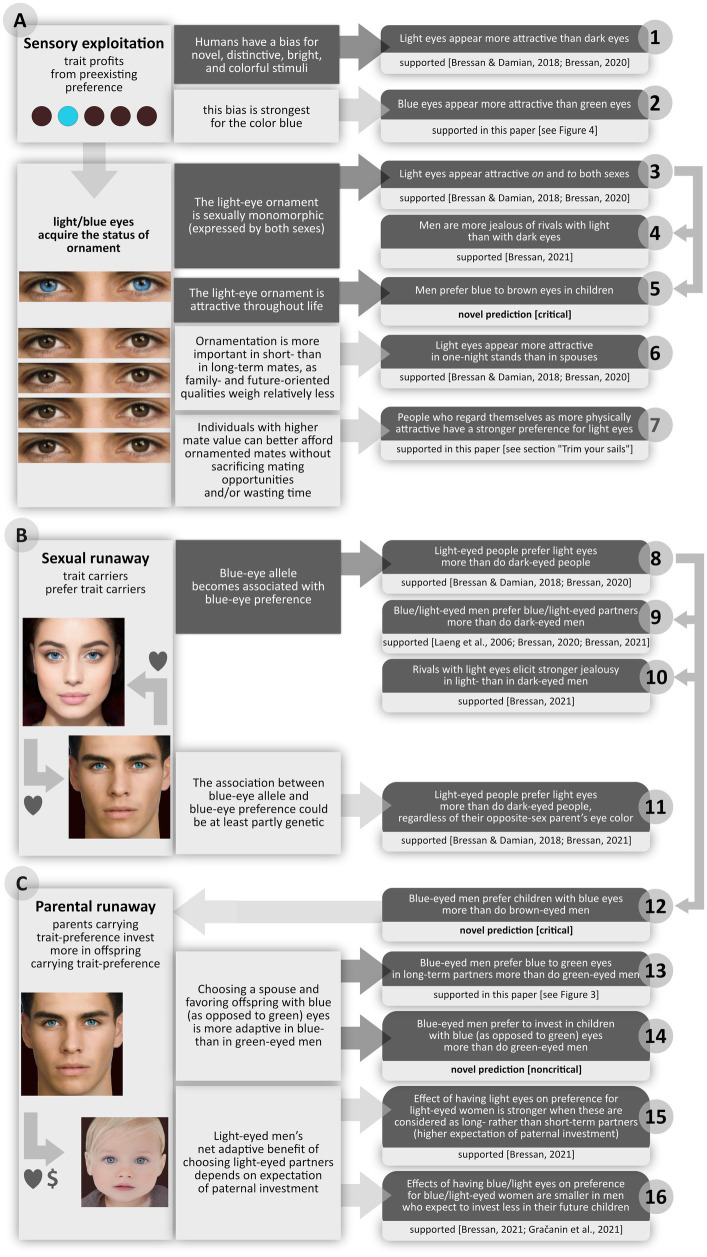
Runaway theory of why humans evolved blue eyes. Left column: mechanisms. Middle column: specific rationales (core ones are on a dark background). Right column: predictions (dark background) and prediction statuses (light background). All predictions refer to individuals of European descent in populations with eye color polymorphism. Predictions that, for lack of relevant data, cannot at present be assessed are provided as independent tests and formulated so as to be fully falsifiable: *critical* predictions are unavoidable consequences of the hypothesis, and would speak against it if they were disproved; *noncritical* ones are based on auxiliary assumptions that are not necessarily true, so their rejection is not a rejection of the hypothesis. As shown by the arrows on the right side, predictions 4 and 5 are special cases of prediction 3; predictions 9, 10, and 12 are special cases of prediction 8.

The theory I advance here (Figure 1) is that our species’ blue eyes are a biological ornament, expressed by both sexes and attractive throughout life. I will contend that blue eyes spread like mad in much the same way as did other useless or inconvenient ornaments, such as the bicolored face of the mandrill or the cumbersome tail of the peacock.

To explain how this sort of embellishments could possibly evolve, Fisher (1915) came up with the notion that a trait and the preference for it can become genetically coupled, on account of the offspring inheriting the trait from one parent (typically the father) and the preference from the other (typically the mother). Such a process would reinforce itself, because the choosiest females (more marked preferences) would pair with the sexiest males (more extreme traits), producing choosier daughters and sexier sons who would do the same, ensuring even sexier and choosier descendants. Over generations, progressively stronger versions of both trait and preference would thus be selected for—unless and until this exponential increase is stopped by counterselection. Note that, although this is the story of how the peacock got its tail, this runaway process implies that trait and preference will increase in frequency, not necessarily that the trait itself will become more elaborate or go overboard (Ryan, 1990). Simply, females who like the ornament better will pair more often with ornamented males than do undemanding females, helping the “preference gene” bind to the “ornament gene.”

This mechanism, known as Fisher’s runaway selection (Fisher, 1930), in no way requires that the heritable male ornament signals “good genes”—a longer life, a cleaner bill of health, a better ability to sire offspring, or other serviceable attributes. A larger attractiveness to the other sex, in and of itself, is enough to ensure a selective edge (Weatherhead and Robertson, 1979). Indeed, females that choose mates who, for some unfathomable reason, seem alluring to them are likelier to produce “sexy sons” who, for the same unfathomable reason, will seem alluring to other females. And as we will see, in the case of blue eyes that unfathomable reason was far from such.

### 2.1 Blue eyes and “sexy sons” (and daughters)

In most animals (albeit not in all species and not all the time: Hare and Simmons, 2019), preferences are expressed by females, the choosers, and ornaments are displayed by males, the courters. However, human mate choice is roughly (Todd et al., 2007) mutual and human eye color is roughly (Bressan, 2024) unisex. Thus we would expect this particular trait-preference correlation to lead to sons *and* daughters who are simultaneously sexy *and* choosy. This is what has occurred in some monogamous birds with mutual mate choice, such as crested auklets, where both sexes showcase, and find appealing, the same feathery headdress (Jones and Hunter, 1993). Indeed, a heritable feature that is “in itself valueless” (Fisher, 1915, p. 187) can set off runaway selection, provided it draws attention and varies within the population—so that individuals who do and do not carry it can be compared with ease. Blue eye color epitomizes such a trait to an outstanding degree: visually salient in itself, it happens to be attached to the most captivating portion (the eyes) of the most informative part (the face) of the human body. Note that the rapidity and extent of runaway evolution are expected to be higher for traits that are under weak natural selection (Lande, 1981); that yield a relatively large variance in sexual preferences (Lande, 1981), and are hence purely arbitrary as opposed to fitness-related; and for which the most preferred genotype is recessive (O’Donald, 1990). Blue eye color meets all such requirements—with the important proviso, whose consequences we will explore later, that their imperviousness to natural selection is scarcely the same around the world.

For runaway selection to take off, both ornament and preference must of course be able to be passed on to the next generation. The heritability of the *ornament*, blue eye color, approaches 100% (Posthuma et al., 2006). As to the heritability of the *preference*, daughters of light-eyed fathers like light-eyed mates better than do daughters of dark-eyed fathers, that is, they echo their mother’s choice (Bressan and Damian, 2018); and sons of light-eyed mothers like light-eyed mates better than do sons of dark-eyed mothers, that is, they echo their father’s choice (Bressan, 2020b). Note that this evidence is consistent with either direct inheritance of preferences for light eyes or sexual imprinting on the light eyes of one’s opposite-sex parent (with the latter appearing more likely: Bressan and Damian, 2018; Bressan, 2020b). Yet importantly, although “inheritance” would be genetic in the former case and achieved through learning in the latter, either mechanism works to the exact same effect—ensuring the transmission of preferences across subsequent generations. (The two mechanisms might also join forces, as mentioned in passing by Weatherhead and Robertson, 1979. For a discussion of their respective merits, see Todd and Miller, 1993; for a discussion of runaways without genetic correlations, see Bailey and Moore, 2012; Rendell et al., 2011; for a model of sexual selection where preference for a trait is acquired by sexual imprinting on the parental image, see Aoki et al., 2001.)

**Figure 2:**
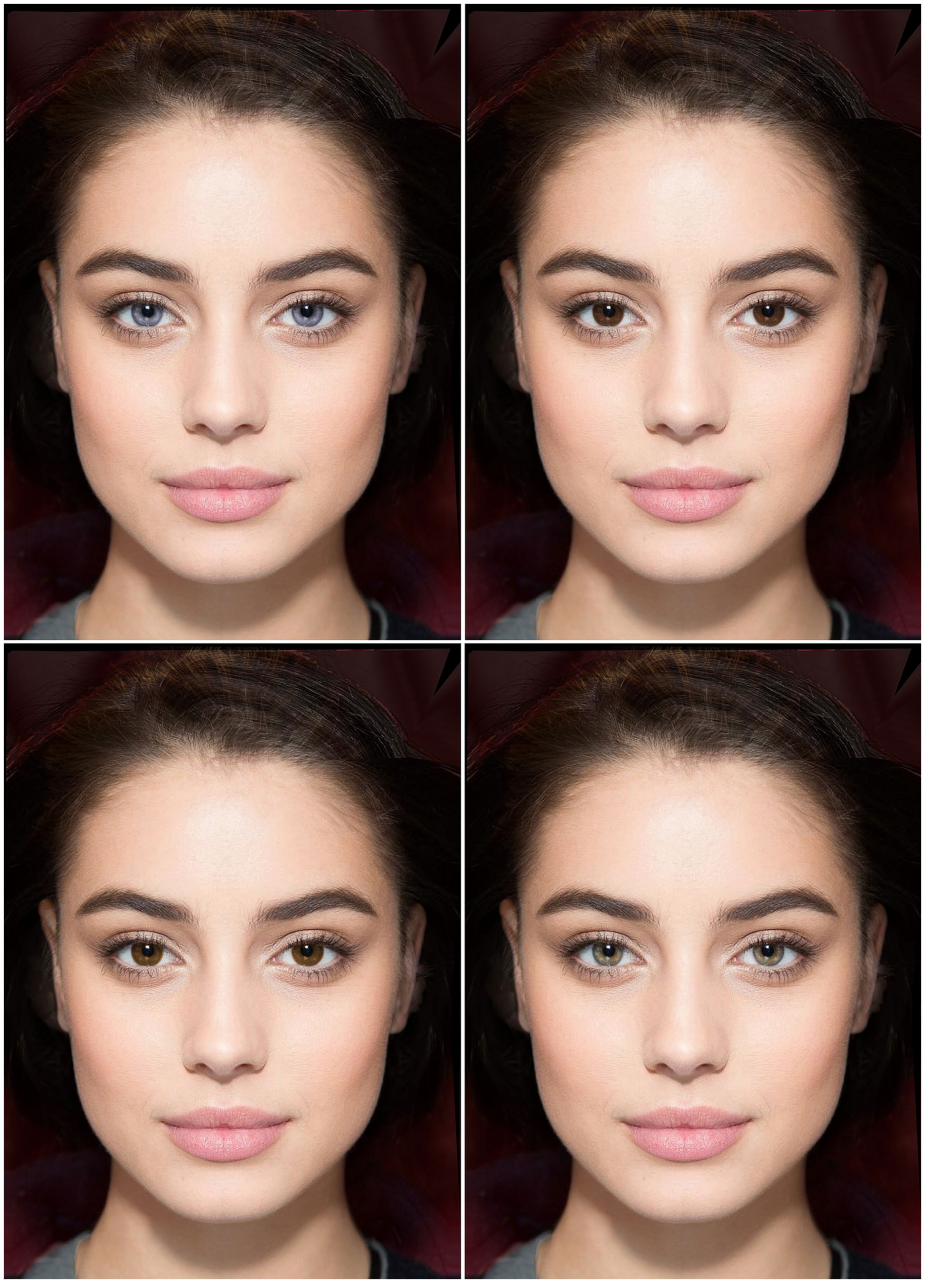
These studies presented pairs of male or female faces differing only in eye color: light (blue or green) vs. dark (brown or dark brown). Top: blue/dark-brown pair. Bottom: brown/green pair. Participants were asked “If you were looking for a long- (short-) term relationship, which of these two people would you prefer?”; they also rated each face’s attractiveness on a 0–10 scale. A description of the methods used in these studies is available online (see Data Availability section). The face depicted here was created digitally for purposes of illustration (Bressan, 2020a); all studies showed photographs of real people whose iris color had been digitally retouched. Image copyright by Paola Bressan.

The idea that, over evolutionary time, the “ornament gene” and the preference should have become statistically associated makes a straightforward prediction. All else being equal, people with light eyes—who bear at least one blue-eye allele, and hence carry the “ornament gene”—ought to prefer light-eyed mates more than do people with dark eyes. This is indeed the case, as shown by studies in which women (Bressan and Damian, 2018) and men (Bressan, 2020b) were asked to judge the facial attractiveness of potential partners varying only in eye color (Figure 2). People tend to be attracted to those who resemble them—leading to “assortative mating,” partners sharing more traits than expected by chance. Thus, it is sensible to ask whether light-eyed people’s predilection for light-eyed mates could be a mere byproduct of this inclination. Assortative mating might be direct, by eye color, or indirect—most plausibly by ethnicity or ancestry, which could create the illusion of assortative mating by eye color just because ancestry and eye color covary (see Bressan, 2021a). And yet, these findings cannot be explained by a simple preference for similar others: individuals with dark eyes did not prefer dark-eyed faces to light-eyed ones. In fact, light-eyed people’s preference for light-eyed potential partners occurred even in the absence of assortative mating for eye color (Bressan and Damian, 2018; Bressan, 2020b).

Therefore, the trait is heritable; the preference is heritable; and the trait-preference association *has* actually arisen. That this association could, in part or in full, be genetic is unnecessary yet far from improbable. First, it is found also in people who are carrying the allele but could not possibly have imprinted on their opposite parent’s eyes, as these are dark (Bressan and Damian, 2018; Bressan, 2021a). Second, a large study on identical and nonidentical twins has shown that several human traits (including hair color; eye color was not tested) are *genetically* correlated with the preference for them (Verweij et al., 2014).

### 2.2 The perks of blue in a world of browns

As mentioned, blue eyes may have happened to lift their early European carriers’ temporary lethargic mood in the wintertime, but this would have been an entirely accidental and not necessarily adaptive side effect. And truth be told, the winter blues explanation accounts neither for the robust association between having and liking blue eyes nor for most of the findings listed in Figure 1—which, as we will see, are all plainly predicted by the runaway theory and compellingly sustained by data.

Here I take the least favorable, most conservative stance possible and assume that blue eyes were indeed, to use Fisher’s words, “valueless”; perhaps even a bit of a nuisance. This does not matter very much: that preferences conferring no survival benefit whatsoever could spread in a population can be demonstrated mathematically (Kirkpatrick, 1982; Lande, 1981). Although Fisher believed that an initial selective advantage is necessary (Fisher, 1915; see also O’Donald, 1980), this has later been shown not to be the case even for traits that *reduce* survival; that are nearly lethal, in fact—provided the initial preference is strong and common enough (Kirkpatrick, 1982; Lande, 1981; Ryan, 1990). And a preference allele for a harmful trait that is not yet present in the population could drift to high frequency, because its bearers would pay no cost if, in the absence of their preferred phenotype, they went about their mating business just like everyone else (Kirkpatrick, 1982; see also Moehring and Boughman, 2019). Once the preferred phenotype turns up, of course, a runaway process could follow very rapidly. This section covers the momentous first encounter of selectively neutral (or slightly disadvantageous) blue eyes with a prebuilt preference that was waiting for them.

I propose that blue eyes were favored because they happened to exploit a natural bias that was already in place—just like the eye-spots on the peacock tail exploited birds’ inborn attention to eyes and the egg-spots on the anal fin of some male fishes exploited females’ inborn attraction to eggs (Egger et al., 2011). Sensory or perceptual biases such as these can arise via selection in nonsexual contexts, or as purely incidental byproducts of how organisms are built (Endler and Basolo, 1998; Enquist and Arak, 1993). And tellingly, animals do tend to respond more strongly to signals that are brighter or more colorful (Ryan, 1990). A brighter-color bias arises inescapably from the simple need to discriminate among different stimuli, emerging as it does in all recognition mechanisms—be they artificial neural networks (Enquist and Arak, 1994) or real animals, such as chicken trained to peck on colored spots (Jansson and Enquist, 2003). Note that blue irides are both brighter *and* more colorful than dark brown ones (Edwards et al., 2016). Even more tellingly, humans (surely those of European descent, but this propensity might be nearly universal: Saito, 2015) show a strong, natural preference for blue (Crozier, 1999); they also react to blue faster than to any other hues (Hurlbert and Ling, 2007). Brownish colors (that is, dark yellows and oranges) are liked the least. Such inclinations may be adaptive, blue possibly standing as a visual signal of clear sky and clean water—and brown as one of feces and rotten food (Palmer and Schloss, 2010).

The hypothesis that blue eyes function as an ornament in humans—at least those of European descent—just like extravagant, colorful tails do in peacocks, makes an obvious prediction and a less obvious one. The former is of course that, other things being equal, potential mates will appear more attractive if they feature blue rather than brown eyes; the bluer—brighter and more colorful: higher luminance, higher saturation—the better. As we will see, this is indeed the case.

The slightly subtler prediction is that blue eyes will look more attractive in extrapair or temporary companions than in lifelong ones. The reason is that the importance of mating ornamentation is larger in sex contexts such as one-night stands and brief affairs than it is in stable relationships like marriage (Li and Kenrick, 2006), where it is adaptively trumped by other, less skin-deep (iris-deep) qualities. This expectation is supported quite nicely too: the preference for light eyes is significantly stronger when individuals are rated as potential partners for a short-term rather than long-term relationship—an effect which is found independently in women (Bressan and Damian, 2018) and men (Bressan, 2020b).

Even if a preference originally tapped into a sensory or perceptual or psychological bias, of course, whether it persists and how, or how fast, it progresses over time ought to depend on its subsequent effects (Dawkins and Guilford, 1996; Endler and Basolo, 1998). For example, a preadaptation to prefer pink eyes to brown ones might also be possible, yet it would carry such a large reproductive cost that the lineage in which pink irides look attractive would promptly go extinct. The increasingly stronger positive selection on the blue-eye allele suggests, instead, that the preference for blue eyes may soon have begun to pay dividends.

### 2.3 My sons are sexier than yours

The sensory exploitation idea implies that blue eyes will be liked (will look “beautiful”) because they are blue, irrespective of the context in which they are encountered. That is, regardless of whether their bearer is a potential mate or a potential competitor; regardless of whether they adorn the face of a man, a woman, or a child. Now, the ancestral brown-eye allele of rs12913832 is essentially dominant over the derived blue-eye one (Edwards et al., 2016). Homozygotes for the ancestral allele have a 99% probability of being born with brown eyes (Sturm et al., 2008); heterozygotes have eyes that, depending on other genes and modifiers, are usually brown and far less often an intermediate shade, such as green or hazel (Walsh et al., 2012); and the eyes of European homozygotes for the derived, recessive allele are nearly always blue, rarely light green (Duffy, 2015; Edwards et al., 2016). Importantly, if neither parent carries the ancestral allele, a child is *extremely* unlikely (1% according to Sturm et al., 2008; close to zero according to Duffy, 2015) to have brown eyes.

Let us then go back to the evolutionary scenario in which part of the population, of both sexes, was bearing both the allele for blue eyes and the preference for blue eyes. Any man who did would have perceived blue-eyed women as more desirable partners; blue-eyed men as more dangerous rivals; and blue-eyed children as more worthy of paternal investment. Indeed, infants’ perceived cuteness and adults’ caretaking motivation go together (Franklin and Volk, 2018; Glocker et al., 2009; see also Hahn and Perrett, 2014). Therefore such a man, first, would have been inclined to create a family with another bearer of the blue-eye allele. Second, among his alleged offspring, he would have invested more in blue-eyed children (likelier, unbeknownst to him, to be his own) than in any brown-eyed ones (almost certainly someone else’s). And third, he would have inadvertently increased further the probability that those blue-eyed children *were* his own by being especially wary of blue-eyed potential rivals—precisely those rivals most liable to infiltrate blue-eyed adulterine children into his brood.

Whether blue-eyed men do like child faces with blue eyes better than the same faces with brown eyes has never been tested: this happens to be a fully falsifiable prediction, and I offer it here as a critical test of my hypothesis. The other two predictions have been supported by a recent study on over 1,000 men (Bressan, 2021a). First, light-eyed men are indeed more attracted to women with light than with dark eyes (similar results have been obtained, albeit in much smaller samples, by Laeng et al., 2006 in Norway and Gračanin et al., 2021 in Croatia); they prefer them more than do dark-eyed men; and this difference is significantly larger when women are presented as potential spouses than as partners for a brief affair. Second, partnered light-eyed men are more jealous of rivals with light than with dark eyes; and their jealousy of rivals with light eyes is stronger than it is in dark-eyed men. This outcome is neatly reminiscent of the finding that, across species, male ornaments can drive reproductive success not only by being attractive to females, but also by intimidating potential rivals (Hare and Simmons, 2019).

A further prediction of the paternal care argument is that, within the realm of light eyes, all effects should apply to blue eyes more forcefully than to green ones. Blue eyes normally imply homozygosis, whereas green eyes (however novel, distinctive, bright, and colorful) do not. Hence a blue-eyed man with a preference for green, rather than blue, eyes fails to help the spread of either the blue-eye trait or the green-eye preference—let alone of their association. In fact, because a spouse with green eyes typically also carries a brown-eye allele, any offspring with brown eyes would not be less likely to have been sired by this blue-eyed father than would offspring with blue or green eyes. Thus, disinvesting in the former in favor of the latter is not going to help the representation of the blue-eye allele in the next generation nearly as much as it would if one’s spouse had blue eyes. And because green-eyed offspring tend to carry a brown-eye allele too, being partial to such children at the expense of blue-eyed ones halves the odds that the blue-eye allele (with its associated green-eye preference) is further passed on. When choosing a wife, then, blue-eyed men should value blue eyes over green ones more than do green-eyed men—to whom this specific argument does not apply.

**Figure 3:**
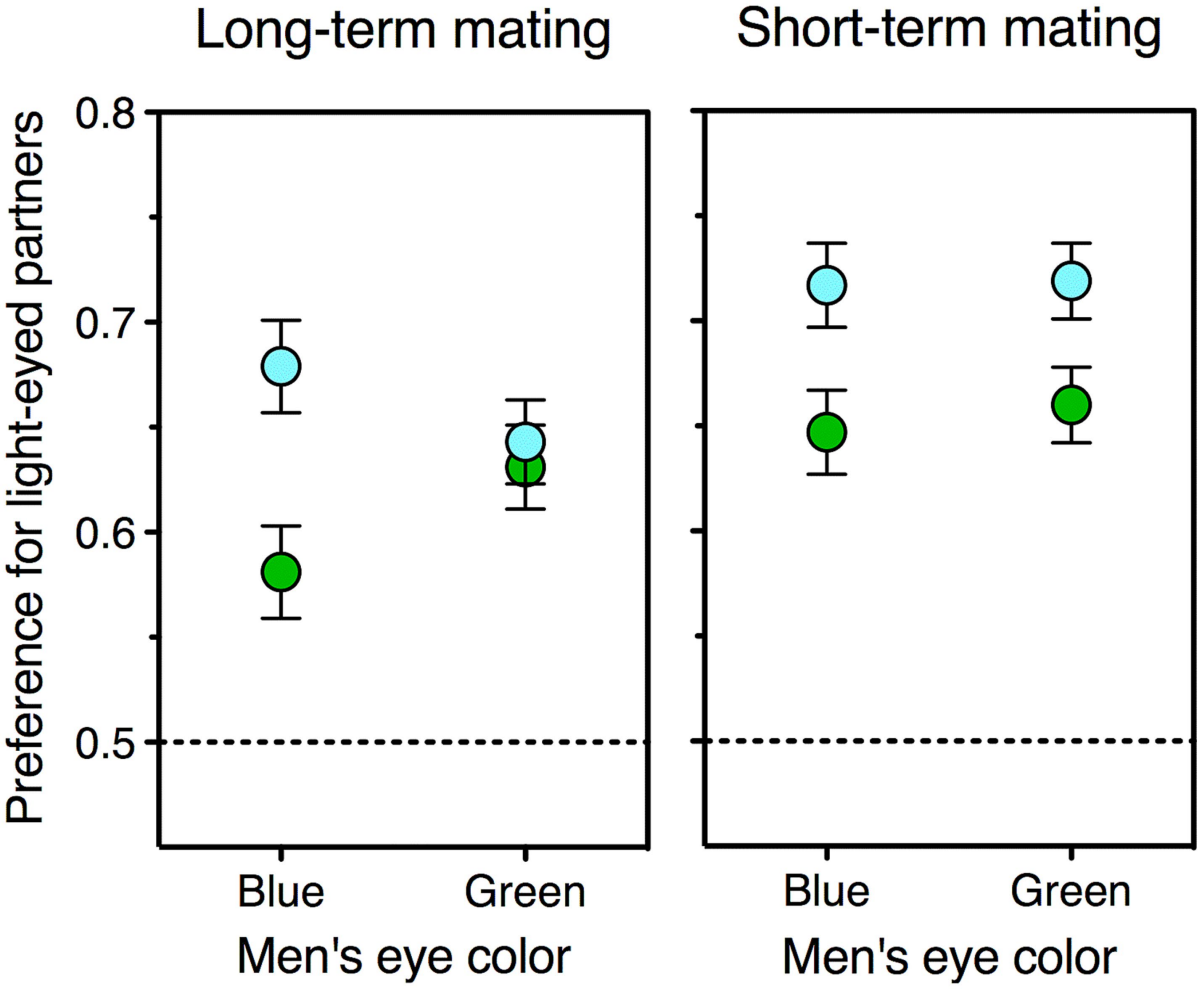
Mean preferences for blue-eyed (blue symbols) and green-eyed (green symbols) female faces relative to identical brown-eyed ones, as expressed by green-eyed and blue-eyed men. Women’s attractiveness as potential partners was estimated for both a short-term (left panel) and a long-term (right panel) relationship. Chance level (no preference) is 0.5; error bars indicate one standard error of the mean. The data refer to all the men in the sample who had blue (*N* = 190) or green (*N* = 236) eyes. Data source: see Data Availability section.

Here I test this prediction, for the first time, using experimental data gathered in my previous work (all of which are publicly available, see Data Availability section; for details on participants, materials, and procedure see Bressan and Damian, 2018; Bressan, 2020b). One advantage of these data is that they were mostly collected in northern Italy—a single-ethnicity area where the entire range of eye colors is far better represented than either further north or further south in Europe (Salvoro et al., 2019). Figure 2 depicts the type of visual stimuli used in these studies. Men’s preference for blue-and green-eyed partners was computed as the proportion of choices of female faces with blue and green eyes relative to the same faces with brown eyes—an index that could range from 0 (light-eyed face is never chosen) to 1 (light-eyed face is always chosen). On these preferences I carried out a repeated-measures ANOVA, with female eye color (blue, green) and relationship type (long-term, short-term) as within-subjects factors, and own eye color (blue, green) as a between-subjects factor. Blue-eyed men preferred women with blue eyes more than did green-eyed men, but only as stable companions rather than as one-night stands—as shown by a significant triple interaction between own eye color, female eye color, and relationship type, *F* (1, 423) = 5.1, *p* = 0.02. Two additional ANOVAs run separately for the two relationship types, indeed, revealed that being blue- rather than green-eyed increased the preference for blue-eyed women as long-term partners, *F* (1, 424) = 8.2, *p* = 0.004, but not as short-term ones, *F* < 1. These results are depicted in Figure 3 and match the prediction quite handsomely.

The observation that blue-eyed men who prefer blue-eyed women may reduce their paternity uncertainty has been made before (Laeng et al., 2006; see also Panzeri, 2003). However, these authors interpreted such a preference as a specific male adaptation: by comparing his alleged offspring’s eye color to his own, a man would be able to identify the child as adulterine in case of a mismatch, and increase his paternity confidence in case of a match. The view I present here is very different. Within the runaway theory, first, this preference is not a specific male adaptation: it just spread along with the blue-eye trait, whichever the trait carrier’s and the chooser’s sex (indeed, light-eyed men *and* women prefer light eyes in a mate more than do dark-eyed men *and* women: Bressan and Damian, 2018; Bressan, 2020b). Men’s preference for “attractively” eyed women would have spread whether or not it raised paternity confidence—much like peahens’ preference for “attractively” tailed peacocks spread even though it did not raise maternity confidence one bit. Second, this preference’s appearance and perpetuation neither require nor imply any eye-color comparisons being made, whether consciously or unconsciously. Simply, due to the association between trait and preference, blue-eyed people (including men) happen to find blue-eyed others (including women and children) more attractive. This would work even if blue-eyed men had no mirrors, no awareness of their own eye color, no matching abilities, and no like-prefers-like rule when seeking a mate or investing in offspring.

Paternal investment affects a child’s future success a good deal, even in times and places where it is not an instant matter of child’s life or death (Geary, 2000; Shenk and Scelza, 2012; Bressan, 2002; López and Ortiz-Rodríguez, 2017). Hence, the partiality of blue-eyed men for blue-eyed children is bound to further increase the next-generation representation of the allele for blue eyes, along with its associated preference for blue eyes—relative both to the allele for brown eyes and to any allele for blue eyes presenting *without* a blue-eye preference. Note that the reproductive advantage accrued to blue-eyed men by their blue-eye preference diminishes as the blue-eye allele rises in frequency in the population, because adulterine children become increasingly likely to have been sired by rivals who are also carrying that allele (see also Laeng et al., 2006). Crucially, however, the advantage never reverses: so long as blue is recessive, it is never better for a blue-eyed man to prefer a dark-eyed spouse, to be warier of dark-eyed rivals, or to invest more in dark-eyed children—not even in a world where blue has become ordinary and brown preciously rare.

Parents who like the ornament better will invest more heavily in ornamented offspring than parents who do not care as much, further hastening the coevolution of ornament and preference. I propose to call this nonsexual evolutionary process *runaway parental selection*. In the special case of ornaments that are passed on as recessive traits, such as blue eyes, parental (here, specifically *paternal*) selection raises the probability of investing in one’s biological offspring, but this need not be always the case, and in general ornament-driven parental selection bears no relationship to paternity uncertainty reduction. The force behind offspring ornamentation—which, chiefly in the form of gaudy colors, has been documented across widely different species—is sibling competition for parental investment in a world of limited resources (Krebs and Putland, 2004). Ornaments can advertise one’s merit and hence personal future prospects, or express temporary need (see Mock et al., 2011; Pirrello et al., 2017). Normally, such ornaments are not related to adult ones (Krebs and Putland, 2004); they disappear as soon as dependence on parents ends (with rare exceptions, e.g., Griggio et al., 2007; Tanner and Richner, 2008); and they work by reliably signaling which offspring is likely to most benefit from parental food and so best increase parental reproductive success. Parents do prefer ornamented offspring, bestowing more food on them, and these grow and survive better than do their unornamented siblings (Lyon et al., 1994; Lyon and Shizuka, 2020). For example, American coot chicks hatching from later eggs, who begin life at a disadvantage relative to their older siblings, happen to be more colorful than their elders and are thus fed more enthusiastically by parents (Lyon and Shizuka, 2020). That these parents indulge the neediest and not the fittest among their offspring hints to the benefits of a flexible, timely appraisal of which ones can be safely neglected (Lyon and Shizuka, 2020; Pagel, 1994).

In humans, on the other hand, blue eye color provides no information about offspring need or survival odds and remains visible and attractive throughout infancy and adulthood—enticing parents from the start and potential mates down the line. Thus one can envisage the evolution of blue eyes as the outcome of two back-to-back, distinct runaway processes or of a single super-runaway fuelled by two separate mechanisms, parental and sexual selection. Indeed, runaway parental selection can be seen as the self-reinforcing coevolution of parental preference and offspring ornament in much the same way as runaway sexual selection is the self-reinforcing coevolution of female preference and male sexual ornament. Note that both processes are driven by social competition between peers—campaigning to be chosen by, respectively, parents and mates—and can hence be pictured as examples of *social*, as distinct from natural, selection (Darwin, 1871; West-Eberhard, 1983). Although both sorts of selection boil down to a reproductive advantage, social selection does not hinge on environmental contingencies but on parents’ or mates’ choices, and relies heavily on what one’s rivals are doing or look like (Darwin, 1871; Lyon and Montgomerie, 2012; Tobias et al., 2012; West-Eberhard, 2014). For instance, the reproductive returns of displaying blue eyes depend on how much they are liked by others and on whether one’s competitors, siblings first and mating adversaries later on, sport blue or nonblue eyes themselves.

Socially selected traits can kick off a Fisherian runaway regardless of whether they are directly associated with mating or not—except that, if they are not, a genetic linkage between ornament and preference is less sure to arise (Bailey and Kölliker, 2019). Yet this is scarcely an issue if the parties are related (West-Eberhard, 1983; Bailey and Kölliker, 2019), and a parental fondness for blue eyes in offspring ensures that both ornament and preference are passed on together just as efficiently as does a female or male fondness for blue eyes in sex partners. The case of blue eyes speaks loudly for the alikeness of the sexual and parental brands of social selection (Queller, 1994; see also Lyon and Montgomerie, 2012; West-Eberhard, 1983, 2014) by showing that both can induce, through notably similar mechanisms, the evolution of notably similar traits: of the very same trait, in fact.

Parental selection makes an appearance in this story even though the success of humans’ colorful eyes would in principle not have required it any more than did the success of peacocks’ colorful tails. Yet on a grander scale, it does make perfect sense that, whenever a heritable ornament is expressed and attractive throughout the lifespan, ornamented offspring will receive larger parental investment—and this unfair advantage will spur or strengthen the runaway.

## 3 Balancing acts

### 3.1 Location, location, location (or: the price of sexiness)

Traits that increase an individual’s attractiveness tend to come at a price. For example, a garish coloration increases one’s conspicuousness to suitors but to predators too. Both trait and preference, then, will keep expanding until the cost of possessing or preferring the trait outweighs the reproductive benefit. In particular, for any population—be they humans or fishes or dragonflies—the optimal color phenotypes and ornaments express a balance between different sources of selection, with environmental conditions (predation risk and other local circumstances, such as temperature) playing a large role (Basolo, 2006; Moore et al., 2021).

The role of the environment is in plain sight here. Barring recent migrations and geographical or historical obstructions, the blue-eye allele’s prevalence diminishes with decreasing latitude in European populations, both ancient (Mathieson et al., 2015a) and modern (Figure 4 in Kidd et al., 2020; see also Walsh et al., 2012). Unlike alleles under recent natural—as opposed to sexual—selection, such as those for lower triglyceride levels or the ability to digest milk in adulthood, the blue-eye one seems to have been already quite frequent thousands of years ago and even *fixed* in Mesolithic hunter-gatherers, genetically close to present-day northern Europeans (see Figure 3 in Mathieson et al., 2015a). Nothing short of impressive, considering that these people were living 8,000 years ago and the successful mutation appears to have occurred just once, possibly a mere few thousand years earlier (Fu et al., 2016). This frenetic pace suggests that blue eyes may well have expanded to near fixation all over Europe, were it not for some counteracting selective forces increasing toward the south. One likely such force—the only one that might be needed—is the detrimental effect of melanin scarcity in regions under stronger ultraviolet radiation. If we look at people with albinism, in whom dearth of pigment is taken to its extreme consequences, truth is that in Africa very few of them seem to survive to old age (Greaves, 2014). In Nigeria, around 90% of albinos are under 30 years (see Hong et al., 2006), and 90% of albinos over 20 have skin cancer (Cervenka et al., 1979). Obviously enough, all hurdles associated with the insults of light (including, most prominently, eye and vision problems) are exacerbated by harsh sun exposure.

There is no denying, then, that light eyes would come at an exorbitant price in regions close to the equator. Still, one may puzzle over why they turned up only in Europe and not at similar latitudes elsewhere, that is Northeast Asia or North America. The “rare/novel color advantage” hypothesis (Frost, 2006, 2014) put this matter center stage by assuming that, being the risky business of hunting in the European steppe-tundra a male affair, the few surviving men must have been surrounded by numerous eager women and hence spoiled for choice. So, in Europe and only there, a selective pressure to show off must have uncharacteristically acted not on men (become choosers) but on women (become courters)—who competed by “diversifying” the color of their eyes and hair.^1^

Even disregarding that women have been far more numerous than men in all populations and at all times during human history (Lippold et al., 2014), the plead that ancestral Europe supplied an ideal propagation environment for the blue-eye mutation drives one to wonder why then, in ancestral Europe, did such mutation not take hold on several separate occasions. Stated another way, the lack of multiple independent blue-eye invasions around the world needs no more explaining than does the lack of such multiple invasions *inside* Europe. The glaring uniqueness of this event (Eiberg et al., 2008) makes the “why not out of Europe” concern rather moot, especially in view of the fact that blue-eye mutations regularly arise in humans as in all sorts of other brown-eyed animals, from goats and horses to koalas (Negro et al., 2017), and via distinct genetic paths (Meyer et al., 2013). Thus, the point is not why a slightly defective eye model failed to supplant the flawless original one in other continents besides Europe, but how it ever managed to do it at all.

This is even more surprising when one considers that, as the allele is recessive, neither the first bearer of a blue-eyed allele nor any of his or her offsprings—or of the first blue-eyed individual’s offsprings, for that matter—would be likely to feature blue eyes. Although such odds are not zero, especially for males (Bressan, 2024; Pośpiech et al., 2016; Sturm et al., 2008), these people’s irides would more plausibly be a lighter brown (Edwards et al., 2016) or an intermediate brownish color. The probability that the hidden blue-eye allele is passed down the line would then halve at each generation, without the mutation ever fully advertising itself. Indeed, blue eyes would have little chance of surfacing or resurfacing unless, before the mutation dies out, two relatives mate with each other *and* both happen to carry the recessive allele *and* both bequeath the recessive, rather than the dominant, allele to a surviving offspring. (Besides, matings between close relatives seem to have been avoided in bands of Paleolithic hunter-gatherers: Sikora et al., 2017.) Putting together the conditional probabilities it is overwhelmingly likely that, just as witnessed in nature, any such allele would rapidly get lost.

I suggest that the one exceptional case in which it did not was linked to one exceptional circumstance in our deep evolutionary past. DNA extracted from remains of ancient Eurasians has revealed that the blue-eye allele was already around 13,000 to 14,000 years ago in places as far apart as northern Italy and the Caucasus (Fu et al., 2016). Hence the carrier of the original mutation must have lived before that, yet later than approximately 54,000 years ago, when a small initial population emerging from Africa founded all extant non-African lineages (Karmin et al., 2015). At the time, Eurasia was marked by abrupt cooling and warming periods—with populations shrinking in size when the ice sheets advanced and re-expanding as they retracted (Posth et al., 2016). And of course, a sharp reduction in the number of individuals (a population bottleneck) ensures that their genetic contribution to future generations will be disproportionately large. Any hidden random mutation a “founding father” happens to carry, say a blue-eye allele, could end up—undeservedly, from a selection viewpoint—in an extraordinarily vast number of people.

Some simulations (Nakagome et al., 2016) have suggested that the blue-eye allele arose earlier than 42,000 years ago, around the time that modern humans arrived in Europe (Benazzi et al., 2011; Lippold et al., 2014); note, however, that such simulations assumed bottlenecks and population expansions that do not match those inferred from ancient DNA, for either maternal (Posth et al., 2016) or paternal (Karmin et al., 2015) lineages. If the mutation is really that old, chances are that it first appeared in the Near East and was then brought into western and central Europe by the ancestors of hunter-gatherers (Günther et al., 2018). A more recent population bottleneck occurred about 23,000 years ago (Tallavaara et al., 2015; Richards et al., 2000), during the last glacial maximum, when the ice sheets reached their greatest extent and northern Europe was rendered inhospitable by ice, storms, and dust. Humans and other animals retreated to so-called refugia, relatively warm pockets in regions such as Iberia, southern France, Italy, and the Balkans, where—reduced in number and isolated from one another—they could have evolved in diverging directions, in response to specific local pressures or by sheer chance (Stewart and Stringer, 2012). Indeed, Europeans were largely replaced by a separate population expanding from one such southeastern refugium (Fu et al., 2016) around the end of the ice age, 17,000 years ago (Bortolini et al., 2021) or earlier (Aneli et al., 2021). And of course, as the climate continued to improve, the abandoned northern lands were recolonized afresh by small numbers of pioneers and then settlers—reindeer and horse hunters (Housley et al., 1997). Any of these genetic bottlenecks would have created the ideal conditions for a blue-eye mutant to leave a deep trace into the future.

Genetic upheavals notwithstanding, note that such a dent is far likelier to have been left by a man than by a woman—simply because, for reasons of starkly lower obligatory investment in energy and time, a male is capable of producing a much greater number of children than a female. Illustrating this basic fact of life, today 16 million men share a particular Y-chromosome sequence derived from one single male, who lived around the time of Genghis Khan and very likely *was* Genghis Khan (Zerjal et al., 2003). Similar examples abound, witnessing the inordinate impact of a few powerful males on the genetic history of the world (Reich, 2018). The extent to which the first bearer of the successful blue-eye mutation shaped the gene pool suggests that he might have been one such powerful male. Note that charismatic, uniquely colored eyes (a lighter or greenish brown if not downright blue: Bressan, 2024; Pośpiech et al., 2016) would hardly be wasted on such a male. Social or even purely sexual privilege—access to a large number of females—would have ensured that very many direct descendants of his did bear the mutation, making it likelier that some of them interbred. Still, whereas it explains why the unrestrained diffusion of a recessive allele would be exceedingly uncommon yet possible, by no means could a bottleneck-founder or Genghis-Khan effect account, on its own, for the evolutionary triumph of blue eyes. Mere genetic drift speaks to virtually none of the empirical findings listed in Figure 1.

Generation after generation, the descendants of the winning mutant carried the allele with them wherever they migrated and mated, throughout Europe and outside of it (Kidd et al., 2020): North Africa, Central and South Asia, and much later America and Oceania. In hot and sunny lands, adaptively, the allele was prevented from being fully expressed. In most people of South Asian ancestry, indeed, two copies of the derived allele make brown eyes lighter and greener, but fail to produce blue eyes (Edwards et al., 2016). And in populations that inhabit regions of low ultraviolet radiation other than northern Europe, such as East Asians (and hence Native Americans: Llamas et al., 2016), the different genetics of eye color makes it hard to see how blue eyes could come about. In the Han Chinese, for example, the rs12913832 chunk of the genome is not associated with eye color at all (Edwards et al., 2016); any mutations affecting iris darkness are primarily, and strongly, involved in skin lightening and have only minimal consequences on the iris itself.

No need, then, to come up with explanations for the failure of colorful eyes to spread to regions of the globe where the blue-eye allele was blocked out by natural selection or never got a chance to express itself. All that is required here is a gene that can mask or unmask the blue-eye allele. An analysis of the eye color records of over 30,000 Italians, which shows that eye color doubly depends on sex—the sex of the parent and the sex of the child—suggests that such a gene sits on chromosome X (Bressan, 2024).

### 3.2 Trim your sails (or: the price of choosiness)

Ornaments can be costly to their bearers but also to their choosers. The stronger the preference for ornamented mates—and hence, the compulsion to discard non-ornamented ones—the higher the price paid in terms of search time and energy. And of course, the higher the risk of remaining unmated (the “wallflower effect”: Kokko and Mappes, 2005). It thus makes good sense to flexibly adjust one’s level of choosiness to one’s environmental and personal circumstances.

I examine here two remarkable examples of such personal circumstances. The first is one’s value on the mating market. Across species, it is the most attractive members that can afford to be choosiest (Buss and Shackelford, 2008; Simão and Todd, 2002), while less attractive ones are forced to scale their aspirations back (Conroy-Beam et al., 2019; Holveck and Riebel, 2010). So the theory predicts that, in the context of a brief affair (where ornamentation’s importance is undiluted by considerations that are only relevant for stable relationships), the preference for light eyes should be smaller in individuals who regard themselves as relatively unattractive. Here I test this prediction for the first time, using the databases described above (see Data Availability section). As it happens, irrespective of whether they are men or women and have light or dark eyes themselves, people whose self-assessed physical attractiveness is below the median (0–6) prefer light- over dark-eyed mates *less* than do people whose attractiveness is above it (7–10), *F* (1, 2240) = 9.39, *p* = 0.002. Or put another way, the more attractive one considers herself or himself to be, the stronger one’s tendency to prefer light eyes in a sex partner, *r* (2656) = 0.07, *p* < 0.0005. The correlation is small, but separately significant for women and men.

The other contingency specifically regards human males. Although blue-eyed men may profit from setting a higher acceptance threshold for brown- than for blue-eyed women, it is easy to see that immoderate fussiness would soon put them at an evolutionary disadvantage relative to their competitors. There must come a point when the costs of lower mating opportunities exceed the benefits of higher paternity confidence. Hence the theory predicts that, in a long-term context (where the presumption of paternal investment looms larger), the preference for light eyes should be smaller for men who do not expect to invest in their children. Even though it has not been tested directly, this prediction is nicely supported by at least two strands of evidence. The first hinges on the fact that men who have received less affective investment from their fathers tend to repeat this pattern with their own children (Jessee and Adamsons, 2018), and thus show a weaker inclination to paternally invest. It indeed turns out that, among light-eyed men, those who felt rejected by their fathers are less interested in light-eyed women and less afraid of light-eyed rivals than those who did not feel rejected (Bressan, 2021a). There is no such effect for dark-eyed men; and maternal rejection never matters.

As to the second line of evidence, blue-eyed men’s preference for blue-eyed women has been shown to be larger in men with a “slower” reproductive strategy (Gračanin et al., 2021)—expressed as higher commitment to long-term relationships, more importance given to mate’s fidelity, and stronger parental investment (Lu et al., 2017). This finding, obtained on 32 blue-eyed and 32 brown-eyed men, would need to be replicated in a bigger sample. Note, however, that it is fully consistent with the theory’s expectations. A reasonable symmetrical prediction, still untested, is that personal or environmental circumstances may modulate not only a man’s attraction toward blue-eyed women but also any biases toward blue-eyed children. Birds do it, after all. Coot parents pick the lucky offspring they will stuff with extra food among those with the reddest/orangest plumage (an ornament that signals relative youth), but only do so when some chicks in the brood have hatched later than others—and hence are, in fact, younger and less able to fend for themselves (Lyon and Shizuka, 2020).

## 4 Hazels, ambers, grays and greens

European eyes come in an assortment of colors, including grays, greens, hazels, ambers, and mixtures or combinations thereof. Some such variations, as we have seen, are purely byproducts of the way the light bounces off irides whose tissue is utterly complex and utterly imperfect. Others appear associated with specific mutated alleles, usually interplaying with one another. In particular, these alleles can have entirely different effects depending on whether they are carried alongside the brown-eye or the blue-eye allele(s) at rs12913832 (Duffy, 2015).

Yet although, clearly, all such derived alleles have been maintained—and some may well have been selected for their effects on eye rather than skin pigmentation (Simcoe et al., 2021)—none of them achieved a success even remotely comparable to that of the blue-eye one. Across 14 European populations, for example, the frequency of homozygotes for the allele associated with green eyes at rs1800407 ranges between 1 and 3.6%; whereas, in parts of northern Europe, the corresponding figure for the blue-eye allele is close to 100% (Kidd et al., 2020). This lackluster performance of non-blue light eyes, which is particularly hard to justify in a “rare/novel color advantage” scenario, is fully predicted by the theory presented here.

**Figure 4:**
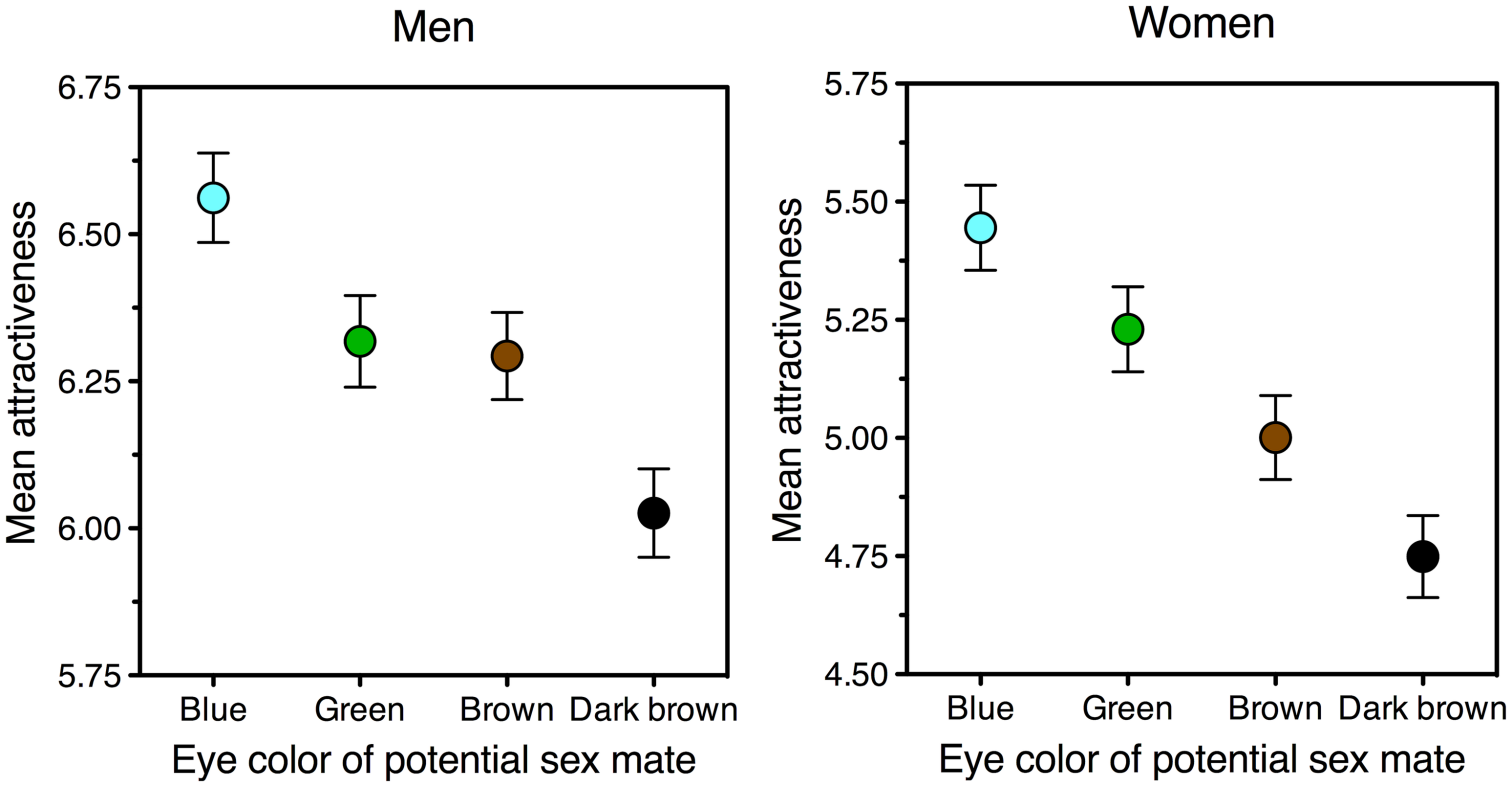
Facial attractiveness of potential sex partners as a function of their eye color, as estimated on a 0–10 scale by men (left panel) and women (right panel). The graphs show that, all else being equal, both sexes like blue eyes best and dark-brown eyes least. As to green and brown, men give them similar ratings (left panel) but prefer green to brown eyes when asked to choose between the two (*M* = 60%, one-sample *t* (569) = 51.9, *p* < 0.0001). The data pertain to the experimental condition in which ratings of physical attractiveness are least biased (a short-term mating context, presented before rather than after a long-term one: Bressan, 2021b). Error bars indicate one standard error of the mean. Data were collected in Italy and refer to all participants (men, *N* = 571; women, *N* = 512) who had blue, green, brown, or dark brown eyes; the preference pattern was the same within each of these subsamples. Data source: see Data Availability section.

Sure enough, light eye colors other than blue could well have been (and still are: Figure 4) preferred to the ancestral dark brown—being novel (Ryan, 1990), distinctive (Jansson and Enquist, 2003), brighter (Ryan, 1990), and, with the possible exception of gray, more colorful (Jansson and Enquist, 2003). However, none would have been able to engender runaway selection, because none are visible to the model’s three relevant selective forces. First, for hues other than blue, any preexisting sensory bias would be weaker and/or restricted to a minority of people. Orangish and yellowish hues tend to be disliked almost as much as browns (Crozier, 1999). Whereas green (albeit only in some shades) could stand a chance, as it often ranks second after blue, its appeal is not nearly as large and universal as that of blue (Crozier, 1999). Second, more importantly, heritability of eye colors other than blue and brown is unreliable and inconsistent, because the alleles associated with them appear to modify their expression depending on other alleles in the genome. As a case in point, the mutations associated with green eyes and mentioned earlier, rs1800407 and rs12203592, can either raise *or* lower the probability of green eyes, depending on whether their carrier has one or two blue-eye alleles respectively (see Table 1 in Duffy, 2015). Hence, somebody carrying such a “green-eye” mutation could be much likelier *or* much unlikelier to have green eyes than somebody not carrying it. This effectively eliminates the chances that trait and preference become statistically associated. And third, lack of trait-preference linkage results in lack of paternal favoritism toward next-generation bearers of the allele. No sexual runaway, no parental runaway.

## 5 Coda: blue eyes as green beards and other interesting implications

In this article I have answered the practical question of why human pale irides persist in many regions of the world even though they carry minor and major health costs. I have done that by proposing a theory of why humans evolved colorful eyes. Or more precisely, a theory of why humans evolved *blue* eyes: from the theory’s angle, amber, gray, hazel, green, as well as light irides of every other description are mere collateral benefits. However sexually appealing and esthetically interesting, they are byproducts of the selection process that ensured blue eyes’ spectacular success.

I have claimed that blue eyes spread by runaway social selection. This particular runaway had on its side not one, not two, but three evolutionary pressures—acting, respectively, on Europeans, on Europeans who carried the blue-eye allele, and on European males who expressed the blue-eye allele. First, blue eyes became potent advertisements for their carriers by exploiting the human sensory and emotional bias for blue. Second, via genetic linkage or parental imprinting or both, the new blue ornament became fastened to the preference for it. And third, ornamented males not only chose—and were chosen by—ornamented females, but also diverted a larger share of resources to the ornamented offspring in their brood. On account of their expressing a recessive allele, these ornamented children were likelier than unornamented ones to be the investing male’s genetic heirs; and because of their father’s favoritism, they were very well placed to hand down their parents’ ornament *and* preference to their own offspring in turn, generation after generation.

It is worth noting that the “ornament gene” for blue eyes, by becoming linked to a “preference gene” that leads its carriers to favor other bearers of the gene for blue eyes, is ultimately helping copies of itself. One is vividly reminded of a classic idea in evolutionary biology, the concept of “greenbeard”: the possibility that a gene recognizes its own copies in other individuals and programs its host to direct benefits to them (Hamilton, 1964). A gene could accomplish that by causing its carriers to display a conspicuous tag, such as a green beard, *and* a behavior related to it, such as being especially nice to other bearers of a green beard (Dawkins, 1976). But of course, this holds too if one gene encodes the tag and a different but tightly associated gene encodes (Haig, 1996, 2020; Jansen and van Baalen, 2006; Sinervo et al., 2006), or simply regulates (Hamilton, 1964; Madgwick et al., 2019), the behavior. It also holds if the nepotistic behavioral trait, rather than being coded by a gene, is acquired early in life, say by imprinting on the parent’s tag (Bressan, 2020b).

It is widely thought that greenbeards, although possible in theory, must be utterly scarce in the real world, as well as unimportant in organisms such as humans (Gardner and West, 2010; West and Gardner, 2013; reviewed in Madgwick et al., 2019). One popular argument for greenbeards’ biological implausibility is that they could easily be outcompeted by cheaters that grow a green beard and thus receive the benefits, but fail to dispense similar benefits themselves. Falsebeards are not a problem for greenbeards of the “ornamental” variety, though, because the association between ornament and preference gets continually reinforced or restored afresh (see the excellent discussion in Pizzari and Gardner, 2012, and the formal mathematical treatment of these issues in Faria et al., 2018). Sure, some individuals with the ornament could pair with individuals without the preference, producing occasional falsebeards (blue-eyed offspring that do not prefer blue eyes); other falsebeards may arise by mutation or, say, failure to properly imprint on the parent (Bressan, 2020b). As they lack the blue-eye preference, however, falsebeards will end up with blue-eyed partners less often than do greenbeards. So falsebeards will be especially unlikely to produce blue-eyed offspring and hence to pass down their falsebeardness (blue-eye ornament without blue-eye preference). They will never be able to drive greenbeards to extinction; we may expect them to remain a rather ineffectual minority instead.

The second common argument against the odds of finding greenbeards in nature is that a greenbeard allele would rapidly spread to fixation (Gardner and West, 2010). At this point it would lose its greenbeard properties, as everybody would have a green beard, eliminating all opportunities for benefits to be bestowed on some individuals but not others. It has been argued that greenbeards could survive only as long as nonbearded individuals keep reemerging by mutation and everybody mates following a like-prefers-like rule (Faria et al., 2018). And yet the fact that an authentic greenbeard such as the blue-eye allele has *not* gone to fixation speaks to a simpler solution, which requires neither of these conditions and proves highly realistic too. The real world is vast and variegated, and the green beards of the real world are likely to meet—sooner or later, someplace or other—some form of counterselection. Gene flow from populations where nongreenbeards are preserved due to local counterselection will be enough to prevent fixation of the greenbeard allele, especially when this is recessive. Indeed, migration would act on a par with mutation as a source of fresh genetic variation (Faria et al., 2018). Importation of genes is bound to occur in all but insulated groups that are also protected from environmental counterselection—such as were, in the case of blue eyes, northern Europe’s Mesolithic hunter-gatherers, among which the derived allele *did* go to fixation (Mathieson et al., 2015b)—and only as long as they remain secluded. Yet the analysis of ancient DNA is revealing that such states of insulation are extraordinarily short-lived in human communities, and that major migrations and population mixtures have been frequent, influential, and often strikingly disruptive during the entirety of our history (Reich, 2018).

If the blue-eye allele is a greenbeard, as it very much appears to be, greenbeards must be everywhere in the animal kingdom. The implications are mostly unsavory. By definition, individuals who carry greenbeard genes recognize and favor one another at the expense of those who do not carry the genes. Thus, the greenbearded are bound to benefit from inside cooperation and form visible or invisible coalitions, while excluding or ostracizing individuals who do not possess the greenbeard gene. Scarcely a bizarre suggestion, as one such real-world case immediately presents itself: the “bluebearded” joining of forces between unrelated male lizards sporting a blue—but not an orange or yellow—throat (Sinervo et al., 2006).

A case that strikes closer to home, if one is not a lizard, is the potentially partisan treatment of embryos by our own placenta. Genes expressed by a mother’s immune cells have all it takes to recognize copies of themselves in placental cells and prompt actions that favor or disfavor the fetus (Haig, 1996). Such genes could easily grant some embryos deeper placental invasion and thus greater access to maternal resources. In so doing, they would bias the mother’s investment toward offspring that inherited them and away from offspring that did not, in impeccable greenbeard style. It turns out, indeed, that a maternally expressed gene, aptly named Medea (for Maternal effect dominant embryonic arrest, but also for the Greek mythological sorceress who murdered her own children out of revenge against their father), does kill embryos without the gene to the advantage of those with the gene (Beeman et al., 1992). Medea exploits a poison/antidote mechanism which ensures that progeny of a carrier mother (producing the lethal poison) survive only if they are themselves bearing a copy of the gene (producing the antidote). This gene is widespread in flour beetles, but it is now being found in other species and happens to be strikingly similar to a human gene (Hudson et al., 1998). Note that Medea machinery spreads swiftly and could provide a vehicle for suppressing or, better, civilizing noxious populations by driving desirable genes into them. For example, it would be entirely feasible to release engineered mosquitoes unable to transmit malaria (Ward et al., 2011).

Just like the greenbeard properties of placental and Medea-like genes express drastic levels of parental favoritism before birth, those of human light eyes extend the potential for offspring discrimination afterwards. Prominently, they hint to the possibility that parents, without their knowledge and entirely beyond awareness, may regard one child more favorably than another on the basis of eye color. Alas, any such bias would be extraordinarily hard to fix, as its underpinning would likely be invisible not only to all involved parties, but to external casual and professional observers too.

On a grander scale, the greenbeardedness of light eyes carries implications of clear social significance, most of which distasteful. The blue-eye allele could in fact lead, unbeknownst to its bearers, to inequitable treatment of others and unfair advantages in social interactions. What is worse, discrimination would be based not on open prejudices or stereotypes or even convenience, but on some irrelevant physical trait. Light-eyed people would unwittingly like, and treat, other light-eyed people better than they like and treat dark-eyed ones. This reminds one forcefully of another color-related unfairness, light-skinned individuals being liked and treated better than dark-skinned ones, for reasons just as capricious and every bit as automatic. Perhaps we should consider taking measures to avoid unjust favoritism based on the color of people’s eyes just like we support policies and practices that avoid unjust favoritism based on the color of people’s skin.

Seeing the sexy-son effect as a special case of the greenbeard effect (see also Faria et al., 2018; Pizzari and Gardner, 2012) has a further surprising implication. All that matters for the greenbeard effect is the sharing of the greenbeard gene; whether carriers are kin or nonkin or have any other genes in common is irrelevant. So, as the blue-eye allele becomes more frequent in the population, it is true that the reproductive advantage of nepotistic blue-eyed men goes down (because “their” blue-eyed children become more likely to have been sired by blue-eyed rivals). From the viewpoint of the allele itself, however, the selective advantage never decreases. The reason is that blue-eyed children bear copies of the blue-eye allele whether or not they are genetically related to the investing father. When investing in adulterine children with blue eyes, fathers are still investing in blue eyes: by hitchhiking on the preference, the allele is *always* helping its own copies. Blue eyes are practical peacock tails, artful parent traps, and finer greenbeards than green beards themselves.

## Data Availability

All data, analysis scripts, and annotated outputs are publicly available via the Open Science Framework and can be accessed at https://osf.io/r3vk2/.

## Footnotes

^1^Note that the runaway theory I propose here dispenses with both the *ad hoc* premises of the “rare/novel color advantage” hypothesis—that courters must have swapped roles with choosers and that sexual selection must have acted only on women.
